# Exploring the relationship between accommodation and intraocular pressure: a systematic literature review and meta-analysis

**DOI:** 10.1007/s00417-024-06565-z

**Published:** 2024-07-22

**Authors:** Giacomo Ambrosini, Silvia Poletti, Gloria Roberti, Carmela Carnevale, Gianluca Manni, Giulia Coco

**Affiliations:** 1 Department of Clinical Sciences and Translational Medicine, University of Rome Tor Vergata, Rome, Italy; 2 IRCCS-Fondazione Bietti, Rome, Via Livenza, 3, Rome, 00198 Italy

**Keywords:** Accommodation, Intraocular pressure, Accommodative task, Near vision, Glaucoma

## Abstract

**Purpose:**

To investigate the relationship between accommodation and intraocular pressure (IOP).

**Methods:**

Systematic literature search and meta-analysis following PRISMA guidelines was conducted on studies analyzing the relationship between accommodation and intraocular pressure. After removal of duplicates, title and abstract screening, full-text analysis was performed to select relevant articles and meta-analysis was then conducted as well.

**Results:**

Of the 1357 records identified, 17 met the selection criteria and were included. Overall, all studies showed that accommodation can influence IOP levels and meta-analysis indicated a significant IOP reduction of 1.10 mmHg (95%CI, -1.77; -0.42) following accommodative stimulus in healthy individuals, albeit with high heterogeneity among studies. Differences in IOP changes between emmetropic and progressing myopic individuals were not significant. Controversial results were obtained in patients with glaucoma with significantly lower IOP fluctuations being noted in eyes with previous trabeculectomy; however, the clinical heterogeneity of enrolled patients among studies made it not possible to combine results. Type of accommodative task, extraocular muscle contraction, head and body position all could potentially play a role in the measured IOP changes with, interestingly, near reading on a smartphone suggesting IOP increase.

**Conclusion:**

Accommodation has an impact on IOP measurements and, overall, determines IOP decrease in healthy individuals. While such variations might not hold clinical significance for individuals in good health, their impact in patients with glaucoma should be considered. Further studies focused on specific components of such relationship are required to elucidate their individual impact and to define their potential role as non-pharmacological strategies to reduce IOP levels in selected patient categories.

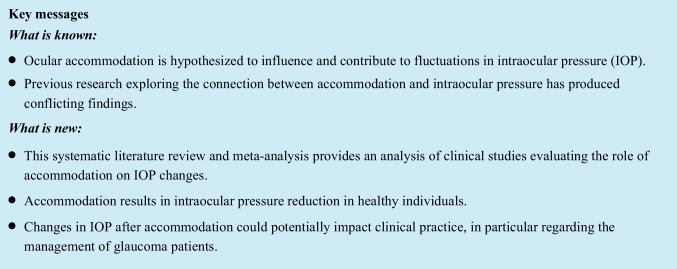

## Introduction

Accommodation is the ability of the eye to modify its refractive power and to bring subjects of interest at different distances into focus [1]. When the eye focuses at a near object, the ciliary muscle contracts, thus reducing the tension in the zonular fibers and allowing the lens to take a more spherical (less flat) shape, enhancing the refractive power of the eye [1].

Intraocular pressure (IOP) levels are determined by a fine balance between aqueous production inside the eye its drainage out of the eye. Aqueous outflow (AO) is thought to be the most important predictor of baseline IOP [2, 3].

The aqueous humor drainage changes according to the accommodative state, being primarily dependent on the uveoscleral pathway during disaccommodation and on the trabecular pathway during accommodation [4]. In theory, during disaccommodation, the ciliary muscle is relaxed, thus allowing a greater aqueous humor flow through its interstitium and downstream. Simultaneously, the ciliary muscle tendons, which attach to the scleral spur and the cribriform plexus adjacent to the Schlemm’s canal, are not pulling on the scleral spur, causing the trabecular meshwork to be slow, at best [5]. During accommodation, the contraction of the ciliary muscle eliminates the space within its muscle bundles; this process squeezes out the aqueous humor already present and offers greater resistance to new aqueous humor inflow. At the same time, the longitudinal fibers of the ciliary muscle, attached to the scleral spur, cause a posterior shift of the spur. This shift enlarges the spaces within the trabecular meshwork and Schlemm’s canal, effectively reducing resistance and enhancing trabecular outflow. Thus, inflow of aqueous appears to be reduced and outflow facilitated [5].

For these reasons, it has been postulated that ocular accommodation may have an impact on IOP levels and contribute to its fluctuations, making it a topic of interest especially in patients with glaucoma.

Previous studies investigating the role of accommodation on intraocular pressure have reported contradictory results. The aim of this review and meta-analysis is to provide results for clinical studies evaluating the role of accommodation on IOP changes thus allowing a better understanding on how different accommodative tasks may influence IOP.

## Methods

**Fig. 1:**
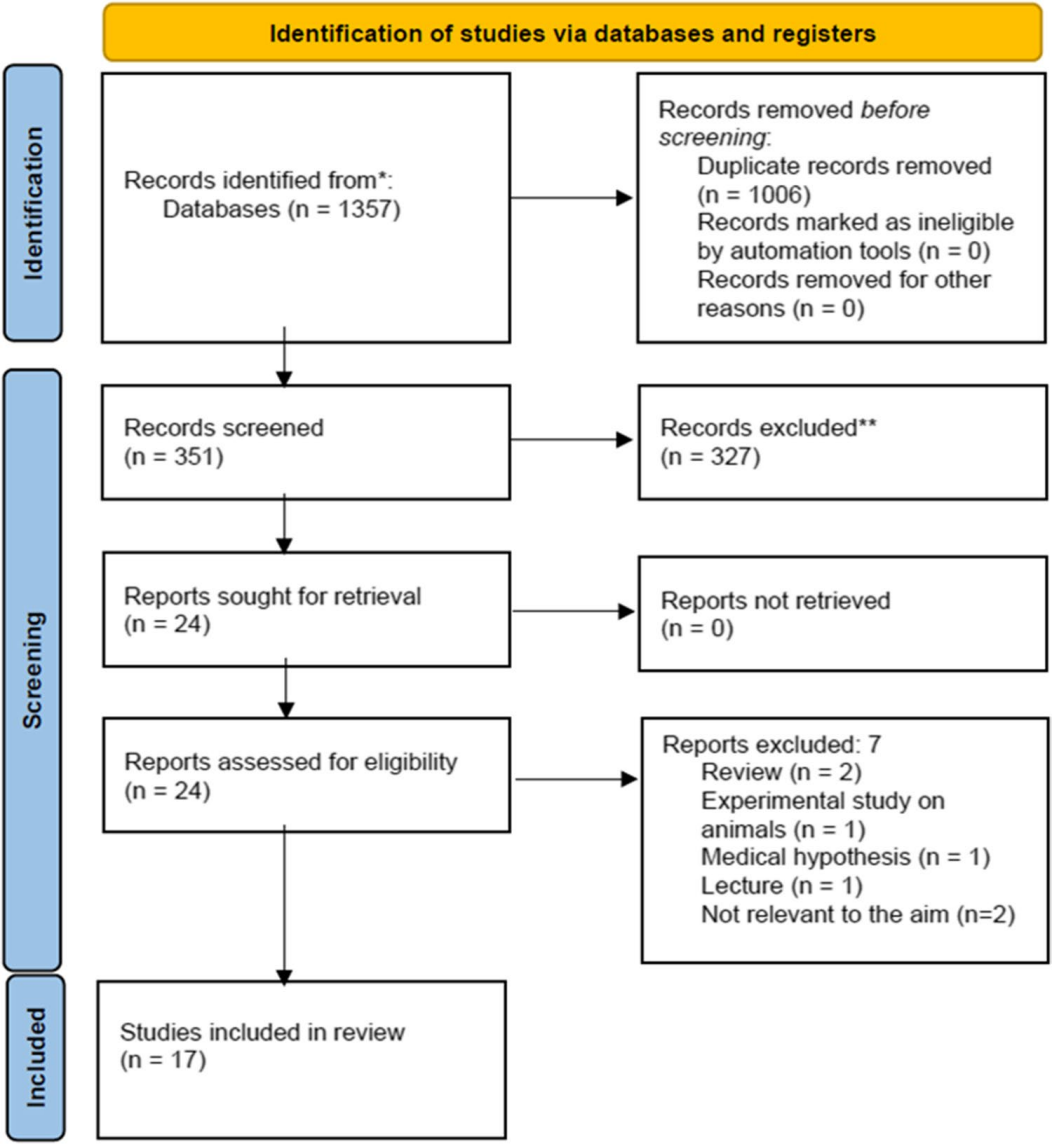
Flowchart of the included studies according to Preferred Reporting Items for Systematic Reviews and Meta-Analyses guidelines (PRISMA)

A systematic literature review (SLR) following the Preferred Reporting Items for Systematic Reviews and Meta-Analyses (PRISMA) guidelines was conducted to determine if accommodation tasks may influence IOP. To identify relevant articles, literature search was performed on April, 1st 2024 in the electronic databases of Ovid Medline, Embase (Ovid), PubMed, Google Scholar, the Cochrane Register of Controlled Trials and the Cochrane Database of Systematic Reviews. There were no restrictions on type of publication, study design or date of publication. The search was performed using controlled vocabulary and test words for ‘intraocular pressure’, ‘IOP’, ‘glaucoma’, ‘primary open angle glaucoma’, ‘accommodation’, ‘accommodative task’, ‘presbyopia’, ‘trabecular meshwork’. Additionally, reference lists of identified articles were manually reviewed to identify any potentially relevant study that may have been missed from the electronic searches. After preparation of the electronic all-data listing, two reviewers (SP and GA) independently reviewed titles and abstracts and identified relevant articles. Studies reporting the effect of accommodative tasks on IOP were included in this review. Studies were excluded if they were review studies, case reports, photo, essays, and studies written in languages other than English. Animal studies and cadaveric studies were also excluded. The same reviewers then reviewed the full texts of the selected articles to confirm their eligibility based on the inclusion and exclusion criteria. Disagreements between the two were resolved and adjudicated by the senior author (GC) (Fig. 1). The articles included in this review are organized within Table 1.

**Table 1:** Characteristics of the included studies

Author (et al.) & year	Study design	Partic ipants	Age Groups	Tonometry	Position	Accommodative task	IOP response	Recovery period/task & IOP	Percentage change
**Healthy subjects**									
Armaly 1958	N/A	7 Healthy	18-25yrs	Mueller electronic tonometry	Supine	Full refraction correction Apparatus with target at 25cm & addition / removal of + 4D lens	C values (aqueous outflow) of the tonograms were greater with accommodation	N/A	
Mauger 1984	N/A	30 Healthy	22–35 yrs Tasks groups (*n* = 10 each)	GAT	Sitting	Fixating the 20/20 line at 20 feet with: • No lens (group#1-control) • -4D lens (group#2) • -1.5D lens (group#3) IOP after 30s and 3.5 min	Group#1-control: -0.35 ± 0.24 mmHg at 3rd measurement Group#2: 30s: -1.32 ± 0.43 mmHg 3.5min: -2.38 ± 0.65 mmHg (ss vs. group#1) Group#3: 30s: -1.15 ± 0.71 mmHg 3.5min: -2.15 ± 0.78mmHg (ss vs. group#1)		Group #1: 3.5min: -2.7% Group #2 3.5 min: -17.2% Group #3 3.5 min: -15.6%
**Ageing**									
Armaly 1961	N/A	10 Healthy	Age-groups (*n* = 5 each) Group#1: 20 -25 yrs Group#2: 45- 55 yrs	GAT	Sitting	Addition & removal of + 4D lens fixating a target at 25 cm Varying degrees of accommodation tested by changing the power of the + lens (0.25D steps)	Overall: -3.58 ± 1.30 mmHg (*p* < 0.01) Group#1: -4.5 ± 1.0 mmHg Time to steady accommodation: 2.7 min Group#2: -2.3 ± 0.78 mmHg Time to steady accommodation: 4.0 min (*p* < 0.01 vs. Group#1) IOP reduction started at 0.25–1.00 D (usually at least 0.50 D) & increased in a range of 0.50–1.50 D	IOP returned to baseline in 90% of cases IOP remained reduced by 0.5–1.0mmHg in 10% of cases	
Blake 1995	Single blind randomize d trial	66 Healthy	20–76 y Group#A (readers) (*n* = 33) Group#B (control) (*n* = 33)	GAT	Sitting	Full refractive correction & presbyopia correction. Read at the nearest point of focus to use residual accommodation Group #A: read for 15–20 min Group #B: look at a distant target (6m)	Group#A: -2.27mmHg • Under 40s: -2.47mmHg • Over 40s: -2.02mmHg (ns) Group#B: -0.56mmHg (*p* < 0.01 vs. group#A)		Group#A: ≅ -14.4% (from graph) GroupB: ≅ -3.8% (from graph)
**Static vs. Repeated accommodation**									
Jenssen 2012	Randomized single- blind study	33 Healthy [SE: ± 2D]	20-29yrs Tasks groups (performed in different order In subsequent days)	GAT & Infrared photorefractor (PowerRef II)	Sitting	Task#1: Focus on a near target (Lang fixation cube No.1) with accommodative demand of 3D for 3 min (static accommodation) Task#2: Alternate focus on a distant and a near target for 3 min (3 s each) (repeated accommodation)	Static accommodation: -1.76 ± 1.17 mmHg (*p* < 0.0001) Repeated accommodation: -2.06 ± 1.48 mmHg (*p* < 0.0001) (ns vs. static; *p* = 0.35)		Static accommodation: -10.7% Repeated accommodation: -15.3%
Stokkerma ns 2020	N/A	31 Healthy [SE: -3.50D to + 2.50D]	Age groups: 20s: 20-25yrs 40s: 40–49 yrs 600s: 60–69 yrs	Pneumoto nometer	Sitting with head on a chinrest	Tasks performed in different order focusing on a 40cm target (iPhone): • 10 min of nonaccomodation (full correction plus + 2–50 lens) • 10 min of accommodation (addition of a -3D lens to simulate accommodation without convergence) • 10 min of alternating accommodation and nonaccomodation (1min cycles) 20min resting period between tasks	Results for the RE: Nonaccomodation: ns IOP change • 60yrs group: -1.6 mmHg (*p* < 0.01) Continuous accommodation: no IOP change (*p* = 0.65) Alternating accommodation: Overall, ss IOP decrease by -0.7 mmHg (*p* = 0.029) • 60yrs: -1.1 mmHg (*p* = 0.028)	Resting period (20 min) IOP returned to baseline	Nonaccomodation: -1.8% Alternating accommodation: -3.9% 60yrs group, nonaccommodation: -10.7% 60yrs group, alternating accommodation: -5.7%
**Stable and Progressing Myopes**									
Read 2010	N/A	32 Emmetropes [SE: -0.5D to + 0.5D] & Progressing myopes [SE: -1.25D to -6D] *Progression* $\ge$ *0.5D/24mo*	23 ± 3yrs Emmetropes (*n* = 17) Progressing Myopes (*n* = 15)	Pascal Dynamic Contour Tonometry	Sitting	Fixation at a near target with accommodative demand of 3D for 2 min	Emmetropes: -1.9 ± 1.4mmHg (*p* < 0.001 vs. baseline) Progressing myopes: -1.8 ± 0.8mmHg (*p* < 0.001 vs. baseline) (ns vs. emmetropes)		Emmetropes: -11.3% Progressing myopes: -10.2%
Yan 2014	N/A	86 Emmetropes [SE: -0.50D to + 0.50D] & Progressing myopes [SE: -12.50D to -0.75D] *Progression* $\ge$ *0.5D/12mo*	Emmetropes (*n* = 40) 26.3 ± 6.5yrs Progressive myopes (*n* = 46) 23.1 ± 10.7 yrs	iCare rebound (between 3-5pm)	Sitting	Full refractive correction and focus on a distant target with a + 3D lens for 5 min to rest accommodation Lens removal and addition of a -3D lens to stimulate accommodation for 3 min Addition of a -6D lens to increase accommodation for 3 min	Emmetropes 3D accommodation: -0.62 ± 2.78 mmHg (ns) 6D accommodation: -0.76 ± 3.22mmHg (p > 0.05) Progressing myopes 3D accommodation: + 0.80 ± 2.28 mmHg 6D accommodation: + 1.02 ± 2.07 mmHg (*p* < 0.01)		Emmetropes 3D accommodation: -3.6% 6D accommodation: -4.5% Progressing myopes 3D accommodation: + 4.9% 6D accommodation: + 6.3%
Liu 2015	Cross- sectional study	318 Emmetropes [SE: -0.50 D to + 0.50 D] & Progressing myopes [-12.50 D to -0.75 D] *Progression* $\ge$ *0.5D/12mo* & Stable myopes [SE: -8.00 D to -0.75 D]	Emmetropes (*n* = 48) 27.6 ± 6.9 yrs Progressing myopes (*n* = 195) 14.3 ± 6.9 yrs Stable myopes (*n* = 75) 27.6 ± 9.2 yrs	iCare rebound (between 3-5pm)	Sitting	Full refractive correction and focus on a distant target with a + 3D lens for 5 min to rest accommodation Lens removal and addition of a -3D lens for 3 min to stimulate accommodation	Emmetropes: -0.39 ± 2.65 mmHg (p > 0.05) Progressing myopes: -0.19 ± 2.16 mmHg (*p* > 0.05) • < 18yrs: -0.40 ± 2.10 mmHg • > 18yrs: + 0.59 ± 2.17 mmHg (*p* = 0.008) Stable myopes: -0.03 ± 1.68 mmHg (p > 0.05) Ns among groups		Emmetropes: -2.3% Progressing myopes: -1.1% < 18yrs: -2.3% > 18yrs: + 3,7% Stable myopes: -0,2%
**Glaucoma**									
Cassidy 1998	Single blind randomize d trial	20 Newly diagnosed POAG	54–77 yrs Group#A (readers) (*n* = 10) Group#B (non-readers) (*n* = 10)	GAT	Sitting	Full refractive correction & presbyopia correction. Read at the nearest point of focus to use residual accommodation Group #A: read for 10 min Group #B: look at a distant target (6m) for 10 min	Group#A: -2.5mmHg Group #B: -0.35mmHg (*p* < 0.001 vs. group#A)		
Ha 2019	N/A	78 Glaucoma [SE: -6D to + 3D] No VF defect in central 20° NTG medically well IOP controlled & NTG who underwent successful trabeculectomy	NTG medical (*n* = 40) 40.9 ± 7.6 yrs NTG TRAB (*n* = 38) 41.1 ± 7.4 yrs	iCare PRO	Habitual posture	Reading/typing the presented sentence on a smartphone for 25 min (IOP measurements at 5, 15, 25min)	NTG medical: 5 min: + 1.6mmHg (*p* < 0.001) 15 min: + 3.4mmHg (*p* < 0.001) 25 min: + 3.6mmHg (*p* < 0.001) NTG TRAB: 5 min: + 1.3mmHg *p* < 0.001 15min: + 1.6mmHg (ns further increase) 25min: + 1.4mmHg (ns further increase)	Resting period (5 and 15 min) NTG medical: 5 min: -6.1% (p < 0.001) 15 min: + 0.1% (*p* = 0.992) NTG TRAB: 5min: + 2.8% (*p* = 0.053) 15min: IOP + 0.2% (*p* = 0.873)	NTG medical: 5 min: + 11.5% (*p* < 0.001) 15 min: + 24.5% (*p* < 0.001) 25 min: + 25.9% (*p* < 0.001) NTG TRAB: 5 min: + 9.4%, *p* < 0.001 15min: + 11.8% (ns further increase) 25min: + 10.3% (ns further increase)
Pakravan 2022	N/A	98 (118 eyes) POAG PACS Matched controls Normal young adults (NYA)	POAG (*n* = 25, eyes = 46): 55.4 ± 14.0 yrs PACS (*n* = 24, eyes = 47): 56.3 ± 13.8 yrs Controls (*n* = 25, eyes = 50): 51.64 ± 11.1 yrs NYA (*n* = 24, eyes = 48): 29.75 ± 3.65 yrs	iCare	Seated with the neck in neutral position	Reading with presbyopic glasses a text at 33 cm (19 Samsung monitor) for 1 h Daylight IOP every 15min	POAG: 15min: -0.98 ± 2.2 mmHg (*p* = 0.004) 30min: –1.17 ± 2.24 mmHg (*p* = 0.001) 45min: –0.8 ± 2.46 mmHg (*p* = 0.032) 60min: –0.63 ± 2.4 mmHg (*p* = 0.081) PACS: 15min: –1.19 ± 2.09 (*p* < 0.001) 30min: –1.02 ± 2.93 (*p* = 0.021) 45min: –1.43 ± 2.89 (*p* = 0.002) 60min: –1.17 ± 2.64 (*p* = 0.004) Controls: 15min: –0.3 ± 1.93 mmHg (*p* = 0.277) 30min: –1.06 ± 2.4 mmHg (*p* = 0.003) 45 min: –1.1 ± 2.46 mmHg (*p* = 0.003) 60min: –0.66 ± 2.76 mmHg (*p* = 0.097) NYA: 15min: –1.29 ± 2.24 (*p* < 0.001) 30min: –1.54 ± 2.35 (*p* < 0.001) 45min: –2.08 ± 2.55 (*p* < 0.001) 60min: –1.75 ± 2.27 (*p* < 0.001)	Focus on a distant object IOP after 15min Group #1: 15min: –0.07 ± 2.56mmHg; *p* = 0.864 Group #2: 15min: –1.04 ± 2.41mmHg; *p* = 0.005 Group #3: 15min: –0.82 ± 2.4mmHg; *p* = 0.019 Group #4: 15min: –2.15 ± 2.67mmHg; *p* < 0.001	POAG: 15min: -6.7% 30min: -8.1% 45min: -5.5% 60min: -4.4% PACS: 15min: -8.5% 30min: -7.2% 45min: -10.1% 60min: -8.3% Controls: 15min: -2.2% 30min: -7.8% 45min: -8.1% 60min: -4.8% NYA: 15min: -9.0% 30min: -10.8% 45min: -14.6% 60min: -12.3%
**Daily Activities**									
Priluck 2020	N/A	15 Healthy [SE: − 0.875D to + 0.125D]	24.3 ± 1.3yrs	iCare TA01i	N/A	Reading a text (2011 MacBook Pro laptop) at 33cm for 20min while listening the text played (near work) IOP every 5min	Near work: 5min: –1.32 ± 0.60mmHg (*p* = 0.15) 10min: –1.72 ± 0.60mmHg (*p* = 0.030) 15min: –1.98 ± 0.60mmHg (*p* = 0.008) 20min: –2.25 ± 0.60mmHg (*p* = 0.002) IOP decreased compared to baseline IOP after 10 min of near work (− 1.60 ± 2.2 mmHg, *p* < 0.05)	Far work: Reading at 520cm (on a 3rd generation 9.7-inch iPad) for 20 min 5min: -1.85 ± 0.60mmHg (*p* = 0.015) 10min: –2.18 ± 0.60mmHg (*p* = 0.002) 15min: –2.38 ± 0.60mmHg (*p* < 0.001) 20min: –1.98 ± 0.60mmHg (*p* = 0.008)	Near work: 5 min: -7.6% 10min: -10.2% 15min: -12.1% 20min: -13.5% Far work: 5min: -11.5% 10min: -13.4% 15min: -14,7% 20min: -12.1%
Baser 2017	Prospective	44 Healthy	20–40 yrs	GAT	Sitting/standing Habitual reading position (majority with a slightly/moderately bowed head)	Reading a near text for 5 min Speaking Carrying a shopping bag for 5 min	Reading: -0.05 mmHg (*p* = 0.188) Speaking: + 0.98 mmHg (*p* < 0.001) Carrying a bag: + 0.99 mmHg (*p* < 0.001)	Resting period 15min: IOP began to decrease 1h: IOP returned to baseline	Reading: -1.1% Speaking: + 6.0% Carrying a bag: + 5.6%
Ha 2018	Prospective comparative case series	39 Healthy	22–38 yrs	iCare PRO	Habitual posture	Reading/typing the presented sentence on a smartphone for 25 min (IOP measurements at 5, 15, 25min) Daylight and low-light condition on consecutive days Baseline IOP at 5 min after daylight/low-light adaption	Daylight: 5 min: + 0.4 ± 2.5 (*p* < 0.001) 15min: + 1.8 ± 2.5 (*p* < 0.001) 25min: + 1.6 ± 2.5 (*p* < 0.001) Low-light: 5 min: + 1.7 ± 2.6 (*p* < 0.001) 15min: + 3.4 ± 2.7 (*p* < 0.001) 25min: + 3.1 ± 2.57 (*p* < 0.001)	Resting period (5 and 15 min Daylight rest: 5 min: + 0.9 ± 2.1% (*p* = 0.220) 15 min: + 0.5 ± 2.2% (*p* = 0.283) Low-light rest: 5 min: -8.1 ± 3.0% (p < 0.001) 15min: -0.3 ± 2.6% (*p* = 0.360)	Daylight: 5min: + 2.0% 15min: + 12.9% 25min: + 11.1% Low-light: 5min: + 12.1% 15min: + 24.7% 25min: + 23.1%
Vera 2020	N/A	24 Healthy [SE -2.0D to + 1.75D]	21.6 ± 1.0 yrs (men; *n* = 12) 22.5 ± 3.0 yrs (women; *n* = 12)	iCare PRO	Sitting or Supine with neck in the neutral position	Reading a text on a smartphone at 30cm for 25 min: • while sitting (at 5, 15, 25min) • while supine (at 5, 15, 25 min) Performed 24–72 h apart at the same time of the day	Sitting: 5min: + 0.9mmHg (*p* = 0.008) 15min: + 0.8mmHg (*p* = 0.008) 25min: + 1.3mmHg (*p* < 0.001) Supine: 5min: + 1.6mmHg (*p* = 0.008) 15min: + 2.2mmHg (*p* = 0.008) 25min: + 2.4mmHg (*p* < 0.001) IOP rise of 1.3 mm Hg and 2.4 mmHg after 25 min of reading in sitting and supine positions, respectively (*p* < 0.001)	Resting period (5min) Sitting: -0.9mmHg Supine: -0.2mmHg	Sitting: 5min: + 5.3% 15min: + 4.7% 25min: + 7.7% Supine: 5min: + 9.3% 15min: 12.7% 25min: 13.9%
Vera 2016	N/A	17 (12 completed) Healthy	24.42 ± 2.84 yrs	iCare rebound Detection of AR	Sitting	Virtual driving session to simulate visual fatigue 2-h drive in a virtual driving environment	IOP and AR decreased (i.e., the accommodative lag increased) after the driving session (*p* = 0.03 and *p* < 0.001, respectively) The nearest distances tested (20 cm, 25 cm, and 33 cm) induced the highest decreases in AR (corrected p- values < 0.05)		IOP decreased by 6.2% after the driving session

AR = accommodative response; GAT = Goldmann applanation tonometry; IOP = intraocular pressure; NTG = normotension glaucoma; PACS = primary angle closure glaucoma; POAG = primary open angle glaucoma; yrs = years

## Meta-analysis [6]

The meta-analysis followed the principles in the Cochrane Handbook and was reported in compliance with the PRISMA guidelines [7]. Studies were selected through the SLR. Nonrandomized studies (NRSs) were included due to the scarcity of randomized clinical trials (RCT) on the topic, despite the expected high risk of bias (RoB) of these studies. The aim of the SLR and meta-analysis was to determine the effect of accommodation on IOP levels in the whole population and in specific subgroups. The following variables were extracted from each included article: author and year of publication, study type, inclusion and exclusion criteria, number of subjects involved and their age, methods of IOP measurements, accommodative task, accommodative demand, duration of accommodation, IOP change after accommodative task. All cases in which multiple tonometry measurements were reported, the measurement closest to 5 min was chosen to enhance consistency among findings. In case of multiple subjects’ groups included in the same study with results reported separately, results from all the appropriate subjects group for each analysis were included. Data extracted from selected articles were reported in a customized Excel (Microsoft Corp.) spreadsheet. The mean ± standard deviation (SD) of IOP change after accommodative tasks were extracted from relevant papers. Whenever the mean ± SD of IOP change was not available, while both the preoperative and postoperative mean ± SD and relative p-value were reported, the mean ± SD of IOP change was calculated in accordance with the Cochrane Handbook for Systematic Reviews of Interventions version 6.4. Standard errors (SE) and 95% Confidence Intervals (95%CI) were calculated following the same source. In case of data only presented as percentage of change or graphically and in case of missing data, the corresponding study was excluded from the pooled analysis of that endpoint.

First, meta-analysis was conducted on all studies including normal subjects of any age who underwent tonometry measurements after accommodative stimulus. Only one among the studies eligible for the meta-analysis included a control group of disaccommodation [8], and similarly, only one eligible study included both static vs. repeated accommodation [9], therefore such comparisons were not possible. Subgroup analyses were performed to control for amount of accommodation and for the role of accompanying convergence and myosis. Results on emmetropic and progressing myopic patients were compared, while non-progressive myopic patients were excluded. Effect of accommodation on IOP in glaucoma patients was also attempted. However, data available from each study included a different subset of glaucoma patients (e.g. open-angle glaucoma, angle closure glaucoma, glaucoma treated with medical therapy or with trabeculectomy), therefore the risk of bias was considered too high to proceed.

All the analyses were performed using STATA 18.0 (StataCorp). Data were pooled using a random effects model. The mean difference was calculated as a measure of effect size for continuous variables. All results were expressed with 95% Clopper-Pearson CI. Statistical significance was defined as *p* < 0.05. To determine heterogeneity among studies, forest plots of study outcomes were examined in their level of consistency. Additionally, the I^2^ statistics were calculated to quantify heterogeneity, assuming values > 50% as indicative of substantial heterogeneity [10]. The maximum-likelihood estimator was used to estimate the between-study variance (τ^2^).

## Accommodation and intraocular pressure in healthy individuals

Starting from the 1950s, some scientists have wondered whether the anatomical changes occurring during accommodation could influence the aqueous humor outflow and consequently intraocular pressure. Most of the first studies were conducted on healthy young adults.

Among the firsts who examined the relationship between accommodation and intraocular pressure were Armaly and Burian in 1958, who conducted a study on 7 young subjects (18–25 years) without ocular pathologies. Measurements were initially made using the electronic Muller tonometer and a specific apparatus was designed through the use of lenses to stimulate and maintain accommodation while avoiding vergence. Visual inspection of the tonograms showed that, almost without exception, accommodation increased the outflow facility. Also, the text refers to the Goldmann equation, which is IOP = (F/C) + P, where F represents aqueous flow rate, C represents aqueous outflow, and P is the episcleral venous pressure. According to the authors, the C value was significantly influenced by the state of accommodation, being greater when the eye was accommodating than when it was relaxed [11].

Subsequently, in 1984, Mauger et al. compared the effects of short-term (30 s) and long term (3.5 min) accommodation on IOP and studied the effect of various amounts of accommodation (1.50 to 4.00 D). Thirty young healthy subjects (22–35 years old) were enrolled, and accommodation was induced through the focus on a distant target and the interposition of negative lenses. Ten of these subjects represented the control group and did not accommodate, while the remaining 20 underwent different amount of accommodation. Repeated tonometry in the nonaccommodative state only reported an IOP decrease of -0.35mmHg at the third measurement. Conversely, accommodation determined a progressive IOP decrease from 30 s to 3.5 min, that was higher at the 3.5 min measurement (-2.38 mmHg vs. -2.15 mmHg in the 4D and 1.5D accommodative task, respectively) [8].

**Fig. 2:**
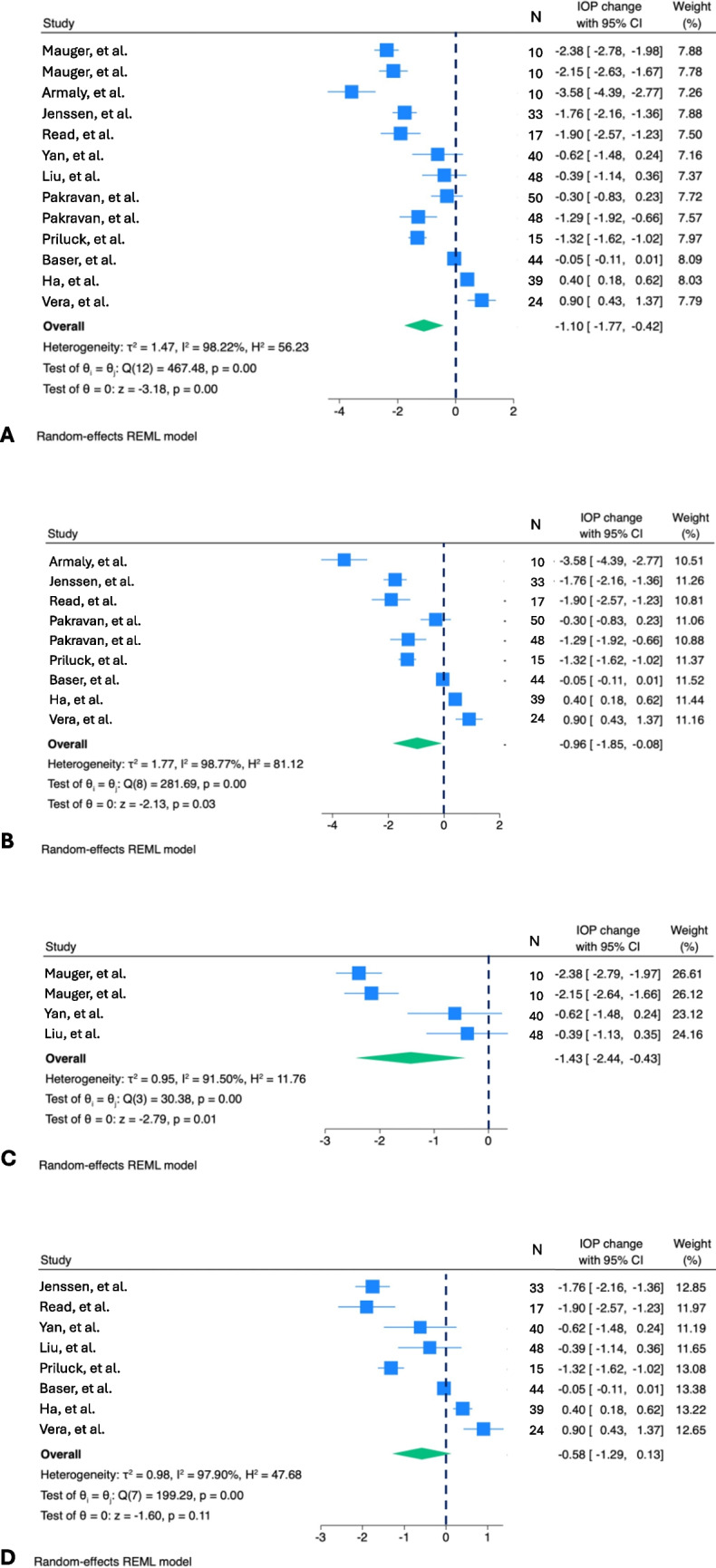
Forest plot showing IOP changes after accommodative stimuli in studies reporting results for normal subjects: **A** All studies reporting IOP changes after accommodative stimuli; **B** All studies reporting IOP changes after accommodative stimuli with eye convergence; **C** All studies reporting IOP changes after accommodative stimuli without eye convergence: **D** All studies reporting IOP changes after accommodative stimuli with accommodative demand of 3 diopters (D). Results are reported as mean and 95% Confidence interval (CI). Heterogeneity among included studies in each analysis was calculated with the I2 statistics, assuming values > 50% as indicative of substantial heterogeneity. The maximum-likelihood estimator was used to estimate the between-study variance (τ2)

From these two studies it emerged that accommodation determined a IOP decrease in healthy subjects, consistent despite two different methods of tonography and induction of accommodative changes. Further studies were conducted aimed at comparing different age groups, accommodative tasks and settings. Overall, the majority of them confirmed some levels of IOP decrease during accommodation in the healthy subject group [9, 12–19], although no change [20, 21] and IOP rise [22, 23] were also sometimes reported (Fig. 2).

**Fig. 3:**
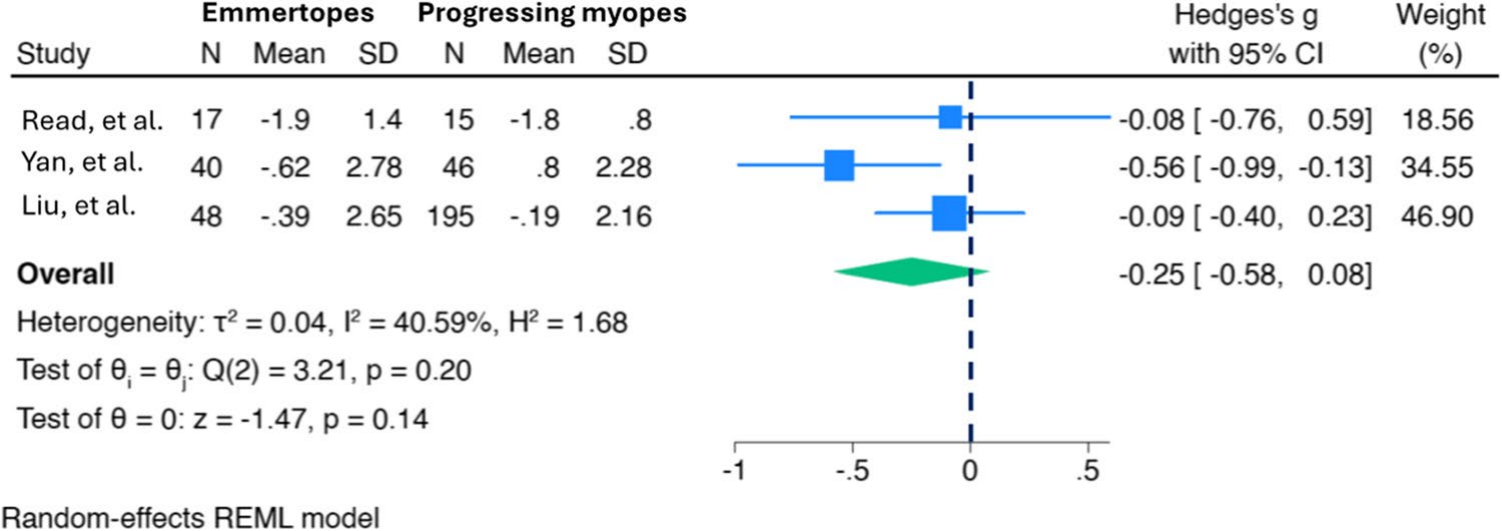
Forest plot showing IOP changes after accommodative stimuli in emmetropes and progressing myopes. Three studies were included with a total of 105 emmetropes and 256 progressing myopes; IOP changes did not significantly differ between groups (-0.25 mmHg, 95%CI, -0.58, 0.08) with heterogeneity level of I^2^ = 40.59% among studies

Meta-analysis of all studies including normal subjects of any age who underwent tonometry measurements after accommodative stimulus revealed an overall significant IOP reduction of 1.10 mmHg [95%CI, -1.77; -0.42] with, however, high heterogeneity among studies (I^2^ = 98.22%; *p* < 0.01). Sensitivity analyses were performed on subgroups of patients (1) performing tasks with accommodative demand of 3D, (2) tasks with eye convergence and (3) tasks without eye convergence. When the restriction criteria of including studies with accommodative demand of 3D was applied, the IOP reduction after the task did not reach statistical significance, while both tasks with and without eye convergence determined significant IOP reductions (-0.96 mmHg, 95%CI, -1.85;-0–08; and -1.43 mmHg, 95%CI, -2.44;-0.43, respectively). Results are shown in Fig. 3.

### Effect of age

Ageing is known to determine a gradual decrease in the maximum accommodative amplitude, reducing from 15 diopters (D) in the early life to 1D at 60 years of age. This physiological process is called presbyopia, and is caused by structural and functional changes in elements of the accommodative mechanism [24].

A few studies examined whether age-related changes in the accommodative mechanism could influence IOP values. In 1961, Armaly and Rubin studied the accommodation-IOP relation in 10 healthy subjects, dividing them into different age-groups of 20–25 and 45–55 years, respectively. Patients were instructed to fixate a target at 25 cm distance and accommodation was evaluated through the addition or removal of a + 4D lens. Various degrees of accommodation were also tested through modulation of the positive lens power. Overall, a significant decrease in the level of IOP was noticed during accommodation, with younger patients showing markedly greater IOP reduction (mean: -4.5 mmHg vs. -2.3 mmHg), significant at the 1% level of confidence [12]. Of interest, they showed that the mechanism of IOP reduction during accommodation was elicited by a minimum amount of accommodation of 0.5D (range 0.25–1.00D) and the range of its maximum utilization was narrow (0.5–1.5D), with most subjects attaining the maximum effect of IOP reduction with less than 2.5D of accommodative power. Such experiments showed that, although the magnitude of accommodation in the older age group was less compared to younger subjects, its level was still equal or greater to the one required to obtain the maximum pressure effect in the younger group. Authors suggested that with ageing, the same amount of accommodation may become less effective in inducing IOP changes, likely due to an earlier selective reduction in the response of the trabecular meshwork to the ciliary muscle contraction [12].

Subsequently, Blake et al. in 1995 conducted a single-blind randomized study recruiting 66 healthy volunteers aged between 20 and 76 years. Participants were instructed either to read for 15–20 min or to look at a distant target. Readers showed a significantly greater reduction in IOP compared to non-readers (-2.27 mmHg vs. -0.56 mmHg; *p* < 0.01) and younger subjects (under 40 s) (*n* = 36) experienced a non-significantly greater IOP reduction (2.47 mmHg vs. 2.02 mmHg in over 40 s; p > 0.05). Such persistence of the IOP fall in the over-forties suggested that the pressure lowering effect due to the contraction of the ciliary muscle was somehow retained while accommodation declined with age [13].

Ageing also showed to have an impact on ocular biometrics. Stokkermans and colleagues found older patients (60–69 years) to have thicker ciliary muscle within 75 μm of the scleral spur, thinner ciliary muscle at 125–300 μm from the scleral spur, narrower anterior chamber angles, shallower anterior chambers and smaller pupils compared to younger patients (20–25 years) (all *p* < 0.01) [21].

### Static versus repeated accommodation

Literature has suggested that the aqueous outflow system may act as a mechanical pump and transient IOP spikes may produce a pulsatile 1-way discharge of aqueous humor into the vascular system [25]. On such basis, frequent and repeated ciliary muscle contractions may induce a similar pulsatile flow, facilitating aqueous humor drainage [9]. The influence of repeated versus static accommodation on intraocular pressure levels was therefore tested.

In 2012, Jenssen et al. conducted a single-blind randomized study on 33 young healthy subjects. Participants were asked to focus on a near target for 3 min in the static accommodation experiment, and to alternate distant and near target focus every 3 s over 3 min, leading to a series of 60 switches between near and distance focusing in the repetitive accommodation experiments. All subjects completed both tasks in different order and in subsequent days. IOP significantly decreased from baseline after both static and repeated accommodation (-1.76 ± 1.17 mmHg and -2.06 ± 1.48 mmHg, respectively; *p* < 0.0001) with no significant difference between tasks (*p* = 0.35) [9].

Later, Stokkermans et al. studied the effect of nonaccommodative, static and repeated accommodative tasks on IOP levels in 35 healthy subjects. Participants were instructed to focus on an iPhone screen at 40 cm distance with either full correction plus a + 2.50D lens (disaccommodation), or full correction plus a -3D lens to simulate accommodation, or to alternate the two tasks within 1-min cycles (alternating disaccommodatoin and accommodation, thus inducing a repetition of accommodation), with every experiment lasting 10 min and a resting period of 20 min between each one. Nonaccommodative and static accommodation tasks did not induce significant IOP variations, while repeated accommodation determined a significant -0.7 mmHg IOP decrease (*p* = 0.028) [21].

Overall, both studies showed significant IOP reduction during repeated accommodation. Different methods to induce accommodation, specifically near-work tasks in the first study and simulated accommodation without convergence though the use of lenses in the second, may potentially be responsible for some differences in the amount of IOP drop and the overall results on static accommodation.

## Accommodation and intraocular pressure in stable and progressing myopes

A number of studies have proposed theories on the association between intraocular pressure and axial globe elongation, suggesting that IOP elevations could lead to posterior sclera stretching and axial elongation, thus playing a role in the development of myopia [26–28]. The association between higher IOP and myopia or myopia progression have reported conflicting results [29–32].

Given the recognized role of the near-work as a risk factor for myopia development and progression [33], and the evidence that near-work tasks can induce transient axial elongation and myopia [34, 35], attention has been paid to investigate the influence of near-work and accommodation on intraocular pressure in patients with myopia. Nevertheless, the precise impact of intraocular pressure on axial length and the development of refractive errors remains inadequately understood and the potential rationale for why the relationship between accommodation and IOP change in myopes should be any different relative to emmetropes is doubtful.

The first study was conducted in 2010 by Read et al., involving 15 progressing myopic [spherical equivalent (SE) range -1.25 to -6.00D and myopia progression of at least -0.50D in the previous 24 months] and 17 emmetropic young adults. Patients were requested to fixate at a near target for 2 min with an accommodative demand of 3D. IOP was reported to reduce significantly in both groups (-1.8 ± 0.8 mmHg and -1.9 ± 1.4 mmHg in myopes and emmetropes, respectively) with no difference between them. Thanks to the use of the Pascal Dynamic Contour Tonometer, they were also able to measure the ocular pulse amplitude (OPA), defined as the difference between the diastolic and systolic IOP over the measurement time (approximately 5 s). Interestingly, myopic patients exhibited significantly lower baseline OPA, and reduced magnitude of OPA changes with accommodation compared to emmetropes, suggesting the presence of some differences in IOP dynamics in myopic eyes [14].

Later, a further study was conducted by Yan et al. Forty-six progressing myopes (SE range -12.50D to -0.75D and myopia progression of at least -0.50D in the previous 12 months) and 40 emmetropes were recruited and instructed to look at a distant target with their full refraction correction. Accommodation was first relaxed with the use of a + 3D lens, and subsequently stimulated with a -3D lens and a -6D lens. Interestingly, intraocular pressure of progressing myopes rose significantly by + 0.80 ± 2.28 mmHg during the 3D accommodation compared to baseline (*p* < 0.01) and compared to emmetropes who, conversely, showed a mild IOP decrease [17]. In both groups, shallowing of the anterior chamber, narrowing of the anterior chamber angle and thickening of the lens was noticed during accommodation (*p* < 0.01) [17].

Lastly, Liu et al. evaluated the accommodation-IOP response in 48 emmetropes, 75 stable myopes and 195 progressing myopes (SE range -12.50 D to -0.75D and myopia progression of at least -0.50D in the previous 12 months). Experimental setting was similar to the study from Yan e al., with distant target fixation with full refractive correction, initial relaxation of accommodation through a + 3D lens followed by an accommodative stimulus induced by the interposition of a -3D lens. All three groups showed little, although insignificant, IOP decrease during accommodation. However, subgroup analysis among progressively myopic patients showed that children (< 18 years old) had a similar IOP decline to others, while adults (> 18 years old) showed a transient IOP increase of + 0.59 ± 2.17 mmHg during the 3D accommodation (*p* = 0.008 compared to children). No impact of the level of myopia was found on the accommodation- IOP relationship [18].

Literature still lacks any firm conclusion regarding IOP changes in myopic patients during accommodative tasks. In fact, among the above-mentioned studies, only the one from Read et al. reported IOP reduction following accommodation in progressively myopic patients; [14] in contrast, Yan et al. showed IOP increase and Liu et al. little and insignificant significant IOP decline which, however, resulted to be a IOP increase in progressively myopic adults [17, 18]. IOP measurements techniques differed between the first and the subsequent two studies (Pascal Dynamic Contour Tonometry and iCare rebound tonometry, respectively), as different were the level of myopia, the definition of myopia progression and the study subjects involved. To note, participants in the latter two studies were Asians, and anatomical and racial differences may have contributed to their findings. Lastly, in the study by Read et al. accommodation was induced with near target fixation, while in the ones from Yan et al. and Liu et al. accommodation was stimulated using a -3D lens while fixating a distant target [9, 17, 18]. Overall, the meta-analysis conducted to compare IOP changes after accommodation in emmetropic and progressively myopic patients, showed non-significant differences with acceptable level of heterogeneity (I^2^ = 40.59%). Results are shown in Fig. 3.

## Accommodation and intraocular pressure in glaucoma

Intraocular pressure is known to be the main risk factor for glaucoma and glaucoma progression, therefore, therapeutic strategies aimed at reducing intraocular pressure values are the primary treatment of choice [36]. Pharmacological and surgical interventions are commonly employed to achieve IOP control. However, several everyday activities and habits may have an impact on the level of IOP [37]. Consequently, researchers are dedicated to determining which lifestyle choices are most effective in lowering IOP levels or minimizing fluctuations [38].

Three studies have been conducted so far to assess the potential variation of intraocular pressure following accommodative tasks in glaucoma patients.

The first study was conducted in 1998. Twenty volunteers with newly diagnosed primary open angle glaucoma (POAG) were instructed to either read at the nearest point of clear focus for 10 min or to look at a distant target. IOP showed significant reduction after reading (-2.5 mmHg; *p* < 0.01), suggesting that accommodation could lower intraocular pressure in eyes with POAG [39].

After approximately 20 years, another study on accommodation involving patients with glaucoma was proposed. Ha et al. examined how reading or writing on a smartphone could impact IOP changes in eyes with glaucoma, and whether IOP fluctuations were affected by previous glaucoma filtering surgery [40]. Seventy-eight patients with either medically controlled normal tension glaucoma (NTG) (*n* = 40) or NTG who had undergone successful trabeculectomy (*n* = 38) were instructed to read and type a text on a smartphone for 25 min in their habitual posture. Both groups showed statistically significant IOP increase at 5 min from the start of the task (+ 11.5% and + 9.4%, respectively; both *p* < 0.001); however, in the medically controlled group, IOP continued to rise during the task up to + 25.9% at 25 min, while no further increase in the IOP was noticed in the trabeculectomy group. In addition, IOP measurements taken after the end of the task showed a IOP drop below baseline levels in the medically controlled group (*p* < 0.001), while a return to baseline levels was noticed in the trabeculectomy group. Such results highlighted the presence of significant less IOP fluctuations in the trabeculectomy group compared to the medication group. Interestingly, in the medication group, a significant correlation between IOP changes and greater neck-flexion angle was noticed [40].

Lastly, Pakravan et al. compared the effect of static accommodation in patients with POAG (*n* = 25), primary angle-closure suspects (PACS) (*n* = 24), matched controls (*n* = 25) and healthy young adults (aged < 40 years) (*n* = 24). Patients were asked to read a text on a 19 Samsung monitor at 33 cm in daylight for 1 h. During the task, all groups showed a statistically significant IOP decrease, with no difference in the mean IOPs and IOP reductions among study groups. This effect was sustained for 15 min after competition of the accommodative task in most groups. From their multivariate analysis, the factor age was associated with greater IOP reduction (*p* = 0.02) [16].

The impact of accommodation on intraocular pressure in glaucoma patients has produced conflicting findings, with one study showing IOP increase during the accommodative task while the other two presenting a noticeable IOP decrease [16, 39, 40]. It is however crucial to highlight two main differences among studies. First, IOP increase was found in normotension glaucoma patients, while studies on POAG or PACS both showed IOP decrease. Secondly, although all tasks involved near-work activities, the specific accommodative task differed greatly, and the neck-flexion angle required to read and type on a smartphone seemed to play a role in the IOP increase during such activity [16, 39, 40]. Due to the clinical heterogeneity among studies in terms of both subjects involved and accommodative tasks performed, meta-analysis was not conducted in this instance due to the expected high risk of bias.

## Accommodation and intraocular pressure in daily activities

Several daily life activities have shown to influence intraocular pressure levels. For instance, activities such as resistance training [41], caffeine consumption [42], and sleeping position [43] have been linked to increases in IOP. In this context, the impact of activities involving the use of accommodation on intraocular pressure levels have also been studied.

As previously mentioned, studies which included healthy subjects and evaluated the impact on IOP of reading a text or reading on a Samsung 19 monitor on IOP levels, usually showed a IOP reduction during the task, although being studies designed for different purposes [13, 14, 16].

Similarly, reading a text on a laptop for 20 min showed to induce a significant IOP drop after 10 min (p > 0.05) remaining lower than baseline also at 15 and 20 min (*p* < 0.05) after the end of the task [15].

Reading, speaking, and carrying a bag, and their influence on the IOP were evaluated by Baser et al., who enrolled forty-four healthy young adults and instructed them to read a text for 5 min on the first day of test, to speak a text aloud the second day and to carry a 5-kg bag for 5 min on the third day. No changes in IOP were observed after reading, while an increase in the intraocular pressure level was noticed after speaking and carrying the bag (both *p* < 0.001) [20].

In contrast, reading and typing on a smartphone screen for 25 min was shown to determine a IOP increase after 5 min (*p* < 0.001), being even higher after 15 min (*p* < 0.001), and then persisting over the course of the 25 min (*p* < 0.001). Both daylight and low-light conditions determined such changes, being more pronounced in the second setting [22]. Subsequent study also evaluated the effect of near reading on a smartphone for 25 min in the sitting and supine position. Results showed that IOP increased while reading in both positions after 5, 15 and 25 min (*p* = 0.008, *p* = 0.008, and *p* < 0.001 respectively). Additionally, IOP measurements while reading in the supine position were significantly higher at 15 and 25 min (*p* < 0.05) [23]. Such results suggested that reclining while reading could lead to more significant increases in IOP.

Lastly, driving was also evaluated, as it being a visual task that mainly relies on the visual system, during which the oculomotor system is continuously stressed to maintain accommodation, convergence, and gaze direction [44]. Vera et al. instructed 12 volunteers to drive in a virtual environment for 2 h. Results showed that both IOP and the accommodative response statistically decreased after the 2-h drive (*p* = 0.03 and *p* < 0.01, respectively) [19].

The above-mentioned results underscore the complex interplay among daily activities, visual demands and IOP dynamics, supporting however the theory that everyday tasks can cause IOP fluctuations.

## Confounding factors in the accommodation – intraocular pressure relationship

Several aspects may contribute to the complexity of IOP changes after accommodation and should be considered in the evaluation of clinical studies. Two main mechanisms are assumed to be involved in IOP fluctuations during near-work tasks, specifically the accommodation-convergence reflex and the external ocular muscle (EOM) contraction. Additionally, head and body posture and psychological stress have also been implicated in IOP changes during everyday activities [22].

### Eye convergence

The accommodation reflex, also known as accommodation-convergence reflex or near reflex, involves multiple mechanisms which are strictly correlated, namely ciliary muscle contraction, pupillary constriction and eyes convergence, this latter one also stimulating EOM contraction [45].

Studies in the literature variably employed different methods to induce accommodation, which could be broadly categorized in utilizing lenses to stimulate accommodation without eliciting convergence or performing near-work tasks (e.g. reading a text) thus triggering the accommodation-convergence reflex.

When lenses were used to induce accommodation while reading or fixating a distant text, thus avoiding the accommodation-convergence reflex, intraocular pressure values in the adult population showed either to remain stable or to reduce [8, 11, 13, 18, 21]. At the same time, the role of accommodation alone was supported by the results from Armaly and Rubin who showed the application of 1% cyclopentolate and 1% atropine to significantly diminish or entirely abolish the IOP reduction following accommodation, while 10% phenylephrine did not result in any alteration in the IOP response, suggesting that pupil constriction does not exert a significant influence on the relationship between accommodation and IOP [12].

In contrast, when near vision tasks were employed, and the accommodation-convergence reflex was thereby triggered, IOP results varied among studies [9, 11, 14–16, 19, 20, 22, 23, 39, 40]. The possible role of convergence on IOP changes was evaluated by Blake et al. through the use of prisms to overcome the need of eye convergence, and the same IOP pressure drop was detected as the one after reading when convergence was allowed, thus suggesting the absence of a role of convergence in the pressure change following accommodation [13]. Meta-analysis also supported this finding, with both tasks with and without eye convergence determining significant IOP reduction (-0.96 mmHg, 95%CI, -1.85;-0–08; and -1.43 mmHg, 95%CI, -2.44;-0.43, respectively) (Fig. 3).

### External ocular muscle contraction

Another mechanism suggested as a potential masquerade for the effects of accommodation on IOP fluctuations is the contraction of the external ocular muscles (EOM). Previous studies showed that EOM contraction led to IOP increase [46, 47]. When using a smartphone, particularly for tasks like scrolling and reading a text, eyes must travel widely, and this requires the eye muscles contraction. Additionally, since hand-held devices can be kept in almost any direction, they may elicit simultaneous vertical and horizontal EOM contraction. These eye movements and the corresponding muscle contraction might be one of the causes of the raise in the intraocular pressure when using a smartphone [22].

### Head & body position

Head position has also been implicated as a possible factor to influence IOP measurements. Malih et al. demonstrated that IOP is significantly higher when measured with neck flexion or extension, with higher values in the neck flexion position [48]. Similarly, Baser et al. proposed that slightly/moderately bowed head might exert a kinking effect on jugular veins, thus preventing the IOP from falling as predicted [20]. In fact, in their study they speculated that head position might have influenced IOP measurements, potentially masking the accommodation-related effects on IOP [20].

In the literature, some studies instructed participants to keep a specific head position by resting their chin on a chinrest during the task, while others asked them to maintain their habitual posture. Interestingly, when head position was controlled for, in most cases IOP tended to decrease during the accommodative task [8, 9, 12, 14, 21]. Conversely, studies in which participants were instructed to maintain their habitual head posture or did not utilize a chin rest yielded varying results [20, 22, 40].

It is noteworthy that Vera et al. demonstrated that changes in intraocular pressure related to reading also depend on body position. They observed higher increases in IOP when individuals, with the neck in a neutral position, were in a supine position compared to a sitting position [23].

### Mental stress

Studies exploring the impact of mental stress on intraocular pressure have indicated that an immediate increase in IOP occurs following exposure to mental stressors, both in healthy individuals and those with open-angle glaucoma [49, 50]. Psychophysiological stress triggers sympathetic input, and the rise in norepinephrine levels, inherent in the sympathetic response, significantly influences the IOP [51, 52]. Because of the multiple stress triggers and prolonged concentration tasks induced, smartphone utilization has been hypothesized as acting as sympathetic stimuli, potentially resulting in an elevation of intraocular pressure [22].

### Diurnal IOP variations

Intraocular pressure levels are known to vary throughout the day, potentially influenced by factors such as ocular biomechanics and circadian rhythms [53, 54]. Nevertheless, only four [17–19, 23] out of the 17 included studies measured IOP changes after accommodation at a specific time of the day, leaving uncertainties on whether the magnitude of IOP change may vary in certain situations (e.g. being greater when the IOP is at its peak or trough). The relationship between IOP changes with accommodation and diurnal variations requires therefore further investigations.

## Discussion

Studies analyzed in the present review and meta-analysis clearly showed that accommodation can influence intraocular pressure levels. Overall, accommodation is able to induce intraocular pressure reduction in healthy subjects [8, 11, 13], with younger adults experiencing greater IOP drop compared to older subjects [12, 13, 21]. No significant differences were found comparing emmetropic and progressing myopic patients, however, literature still lacks of a clear understanding on how accommodative tasks can influence IOP in stable myopic and glaucoma patients and in daily life activities.

The heterogeneity among studies in the literature resulted to be high in most instances, and several reasons can be responsible for this finding. First, IOP assessment measurements differed greatly among studies, being Muller tonometry, Goldmann applanation tonometry and iCare pneumotonometry all variably used. However, within study measurements were made with the same instrument, likely making trustworthy the variations in pressure readings in each individual study. Additionally, methods used to stimulate accommodation were highly variable, being either lens-induced or near-work-induced with different implications in the accommodation-convergence reflex stimulation. However, both previous literature and present meta-analyses suggested the absence of a significant role for eye convergence in the pressure change following accommodation [13]. Furthermore, the impact of body position and psychological stress during daily activities have proved to play a role, and even reading on different devices and in different positions seemed to yield distinct effects [15, 22, 23, 40]. Lastly, another potential confounder could be the variability in the accommodative response induced by the specific task in each study. First, the accommodative demand was not the same throughout studies (Table 1); secondly, only one study directly monitored the accommodative response [9], while all the others assumed an accommodative response matching the presented accommodative stimulus, although without any direct measurement being made. Intraocular pressure variations after accommodation might not hold clinical significance for individuals in good health. Nevertheless, it has been suggested to consider the impact of such fluctuations in glaucoma patients and to potentially use home-based accommodative tasks to reduce intraocular pressure. However, the true meaningfulness of this relationship for most glaucoma patients is hampered by the mean age affected by the condition, that often coincides with the presbyopia age [55] and further studies are required to better explore this condition. Understanding the relationship between accommodation and IOP in patients with juvenile glaucoma could be beneficial, as healthy young patients experience the greatest IOP reduction with accommodation. Activities or interventions that induce accommodation might influence IOP levels, presenting novel therapeutic strategies to control IOP in these young patients, helping to prevent further optic nerve damage.

## Conclusion

In conclusion, there is a link between accommodation and intraocular pressure levels, and healthy individuals experience IOP decrease while accommodating. However, several factors contribute to this dynamic and a full comprehension of how each factor might influence IOP, together with if specific clinical conditions may experience different accommodation-IOP change relationships is still unclear. Further research with better designed and controlled studies is necessary to isolate the individual impact of each single player of this relationship and formulate strategies to make the most of daily activities in IOP regulation.
